# Natural killer cells in neuroblastoma: immunological insights and therapeutic perspectives

**DOI:** 10.1007/s10555-024-10212-8

**Published:** 2024-09-18

**Authors:** Magdalena Rados, Anna Landegger, Lukas Schmutzler, Kimberlie Rabidou, Sabine Taschner-Mandl, Irfete S. Fetahu

**Affiliations:** 1 St. Anna Children’s Cancer Research Institute, Vienna, Austria; 2 Medical University of Innsbruck, Innsbruck, Austria; 3 Department of Otorhinolaryngology - Head and Neck Surgery, Medical University of Innsbruck, Innsbruck, Austria; 4 Department of Medicine, Hospital of the University of Pennsylvania, Philadelphia, USA; 5 Department of Neurology, Division of Neuropathology and Neurochemistry, Medical University of Vienna, Vienna, Austria; 6 Comprehensive Cancer Center, Medical University of Vienna, Vienna, Austria

**Keywords:** Neuroblastoma, NK cells, Immunotherapy, Adoptive NK cellular therapy, CAR-NK cells, Anti-GD2 antibody

## Abstract

Natural killer (NK) cells have multifaceted roles within the complex tumor milieu. They are pivotal components of innate immunity and shape the dynamic landscape of tumor-immune cell interactions, and thus can be leveraged for use in therapeutic interventions. NK-based immunotherapies have had remarkable success in hematological malignancies, but these therapies are met with many challenges in solid tumors, including neuroblastoma (NB), a childhood tumor arising from the sympathetic nervous system. With a focus on NB, this review outlines the mechanisms employed by NK cells to recognize and eliminate malignant cells, delving into the dynamic relationship between ligand-receptor interactions, cytokines, and other molecules that facilitate the cross talk between NK and NB cells. We discuss the immunomodulatory functions of NK cells and the mechanisms that contribute to loss of this immunosurveillance in NB, with a focus on how this dynamic has been utilized in recent immunotherapy advancements for NB.

## Introduction

Natural killer (NK) cells are hardwired innate lymphoid cells that play a primordial role in recognizing and eliminating stressed cells, including infected or transformed cells, in absence of prior immunization [1–3]. This unique ability of NK cells, which confers them with cytotoxic capacity independent of antigen specificity, positions them as primary effectors in the immediate response against infections and cancers [4]. The cytotoxic activities deployed by NK cells are strictly regulated by an array of activating and inhibitory signals delivered by a multitude of receptors expressed by NK cells, which in turn can sense whether a cell in close vicinity expresses corresponding ligands, ultimately triggering NK cell activation and targeted cell killing [5–7]. The current backbone of anticancer immunotherapies involves tumor-specific CD8 + T cells, which are the most powerful effectors against neoplastic transformations [8]. Additionally, use of immune checkpoint inhibitors to enhance the T cell function [9] along with transfer of genetically modified or synthetic receptors (chimeric antigen receptor, CAR) in CD8 + T cells [10] have revolutionized treatment of hematological malignancies and personalized cancer treatment options [11]. Unlike CAR T cells, where a bespoke manufacturing process is needed for each patient to prevent life-threatening graft-versus-host disease (GvHD) and avoid rapid clearance by the host immune system, NK cells are devoid of T cell receptors, allowing for allogeneic transfer [12–14], thus acting as a promising “off-the-shelf” immunotherapeutic modality. Moreover, with current technological advancements, isolation and expansion of NK cells can be readily achieved through various methods, such as isolation from peripheral and umbilical cord blood samples, and through differentiation of pluripotent stem cells into NK cells [15]. In comparison with the personalized CAR T cells, the ability to mass produce NK cells could prove to be extremely advantageous from both, a time and a cost perspective, ultimately improving patient access to these therapies.

Neuroblastoma (NB) is a highly aggressive pediatric tumor of the sympathetic nervous system with a mortality rate of > 50% in the high-risk NB group [16, 17]. Immunotherapy has been met with challenges in this population, and while NB etiology is multifactorial, a large part of the nonresponse is believed to be due to the tumor microenvironment (TME) [18, 19]. The advent of single cell technologies has allowed us to characterize the TME in NB as lowly immunogenic, which is in part due to the low mutational burden of tumor cells [19]. The TME in primary low-risk NB is marked by increased T and NK cell infiltration, which are linked to favorable clinical outcome [20]. The effectiveness of NK cells against NB destruction is further hampered by T cells that express immune checkpoint blockade molecules, along with stromal and myeloid cells that contribute to reduced infiltration and immune response [18, 19, 21]. Therefore, strategies aimed to alleviate this immunosuppression and augment NK cell function are emerging as platforms for new treatment regimens for NB. This can be achieved in various ways: activation by cytokines or stimulation of innate immunity, arming NK cells with chimeric antigen receptors, or biologic therapeutics that allow for NK receptors to link with proteins or ligands expressed on tumor cells. Application of NK cells in preclinical and clinical settings, in the context of NB have shown promising results [22–24], however, the major limitation that persists is the lack of long-term immunological memory.

A leading immunotherapeutic strategy in NB utilizes antibodies against tumor-associated disialoganglioside GD2 [25]. The Fc portion of anti-GD2 antibodies, which are bound to NB tumor antigen, are recognized by NK cells, ultimately exploiting the innate capacity of NK cells to eliminate tumor cells via antibody-dependent cell-mediated cytotoxicity (ADCC) [26]. Several recent studies have evaluated the use of immunotherapy targeting tumor-specific antigens, such as GD2 and/or programmed death-ligand 1 (PD-L1), in relapsed/refractory NB [27, 28]. Dinutuximab, a chimeric murine-human GD2 antibody developed by combining murine IgG3 mAb 14.18 fragments with human IgG1 Fc fragments in SP2.0 cells has been surpassed by Dinutuximab β produced in CHO cells, which offers superior ADCC at lower doses and metastasis suppression in vivo [29]. Efforts to enhance GD2 antibody-mediated immunotherapy have progressed with hu14.18K322A, a humanized Dinutuximab variant with a K322A substitution in the Fc region, which reduces neurotoxicity [30, 31]. Additionally, Naxitamab, another humanized (IgG1) anti-GD2 antibody (hu3F8) has demonstrated superior ADCC and clinical efficacy in combination with granulocyte macrophage colony-stimulating factor (GM-CSF), gaining approval by Food and Drug Administration in 2020 for treatment of high-risk NB [32]. This treatment modality has resulted in significantly improved survival rates for NB patients and has become the standard treatment regimen [33–35]. This form of immunotherapy can also be combined with other standard chemotherapy agents, with good treatment effect [35–38]. Investigations are currently ongoing to optimize the longevity and potency of therapeutic outcomes by enhancing the expansion and activation of NK cells, as well as testing combinatorial treatment with other immunomodulatory agents and/or conventional treatments [38–40].

Here, we review the mechanisms of NK cell recognition, activation, and effector functions in response to NB cells. We discuss how these unique properties can be leveraged in NK immunotherapies, highlighting the opportunities and challenges in exploiting NK cells for NB treatment.

## NK cell target cell recognition and prognostic value in NB

Historically, NK cell-mediated cytotoxicity was described as non-specific [41]. However, it is now well-established that NK cells are governed by a complex array of germline-encoded inhibitory and activating receptors and immune mediators, such as chemokines and cytokines that ensure selective targeting of cells [42–45].

### NK cell cytotoxicity and activation

**Fig. 1:**
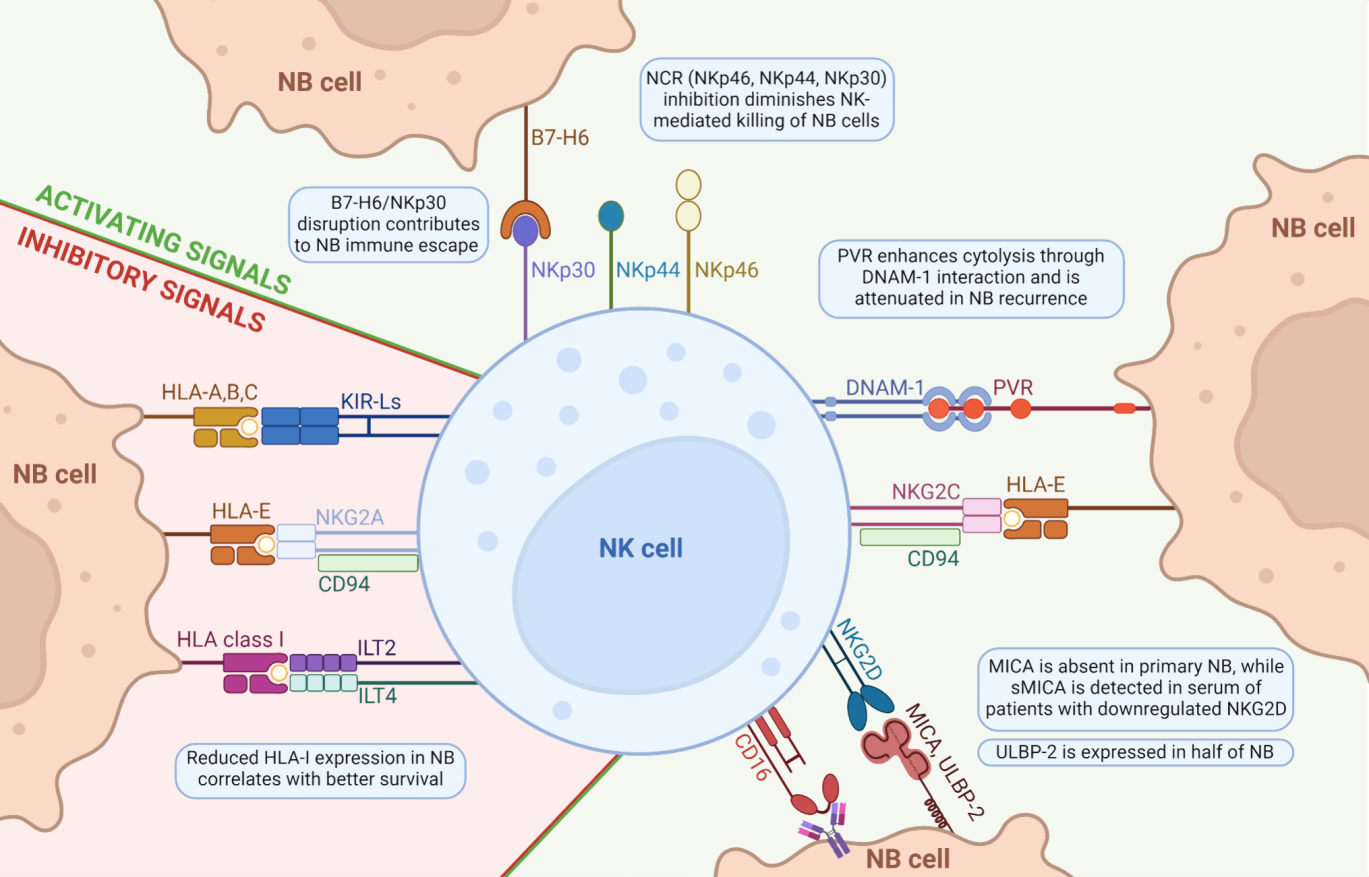
Multifaceted mechanisms of NK cell activation and inhibition in NB. Illustrated in a clockwise direction beginning from the top left: The activation of natural killer (NK) cells is facilitated through natural cytotoxicity receptors (NCR: NKp46, NKp44, NKp30) where aberrant expression of these receptors is linked to immune evasion in neuroblastoma (NB). The DNAM-1-mediated NK activation is initiated through binding to PVR and is attenuated in NB recurrence. Both CD94/NKG2C and NKG2D, members of the NKG2 family, transmit activating signals through engagement with distinct ligands: CD94/NKG2C binds the HLA-E, while NKG2D interacts with MICA and ULBP-2, with ULBP-2 showing a higher prevalence of expression in NB. Additionally, CD16-mediated activation represents another pathway through which NK cells are stimulated in the NB TME. Conversely, inhibitory signals are conveyed through interactions involving inhibitory KIR receptors (KIR-L), CD94/NKG2A, and ILT2/4 with members of the HLA-I family. Notably, reduced expression of HLA-I on NB cells correlates with enhanced patient survival. Figure was created with BioRender.com

The NK cell target recognition system is strictly balanced by activating and suppressing signals, which operate in an integrated fashion where an inhibitory state can be overcome when a strong activating stimulus presents itself. In normal settings, this orchestrated balance allows for effective immunosurveillance [44, 45]. Upon activation, NK cytolytic activity is mediated by the release of cytolytic granules containing perforin and granzymes, ADCC, and death ligands, such as Fas ligand and TNF-related apoptosis-inducing ligand (TRAIL), leading to target cell apoptosis [46]. In the TME, including in NB, this system is disrupted, hindering the killing mechanisms mediated by NK cells (Fig. 1). This, in turn, causes downstream perturbations in receptor expression, signaling pathways, as well as effector functions [47].

NK cells identify major histocompatibility complex (MHC) class I molecules through natural killer receptors (NKRs), suppressing their cytotoxic activity via immunoreceptor tyrosine-based inhibitory motif (ITIMs) signaling [48–52]. Human NK cells exhibit a diverse range of inhibitory NKRs, including inhibitory killer immunoglobulin-like receptors (KIR-Ls), which primarily engage with specific allelic groups of HLA-A, HLA-B, or HLA-C molecules [53, 54]. The C-type lectin superfamily, encompassing CD94 and NKG2A receptors, specifically identifies the HLA-E class I molecules, whereas leukocyte immunoglobulin-like receptors, ILT2 and ILT4, are known for their broad recognition and interact with numerous MHC class I ligands [55]. Stressed, virally infected, or malignant cells lose their protective MHC class I expression, and this is commonly accompanied by de novo ligand expression recognized by activating receptors on NK cells, ultimately resulting in NK-mediated killing [56, 57]. NK cell activation is not exclusively governed by MHC class I expression, as these cells can effectively eliminate target cells with normal MHC class I levels, likely due to high levels of activating ligands that override inhibitory signals. However, the role of MHC class I is critical, as various cancer types have evolved to evade T cell response by downregulating MHC class I expression [58], which provide NK cells with a unique attribute that can be leveraged when considering treatment approaches, particularly for tumors that are characterized by resistance to current antitumor CD8 + T cell therapies [59, 60].

A study comparing primary NB to healthy adrenal medulla reported that undetectable MHC class I antigen levels in NB tumors were due to compromised antigen presenting machinery involving a host of proteins (zeta, tapasin, TAP1 or TAP2, HLA class I heavy chain, β2 microglobulin, LMP2, and LMP7) [61]. Another study highlighted that low expression of MHC class I was due to reduced levels of NF-kB and IRF1, with the level of expression via immunohistochemistry staining corresponding to low-risk versus high-risk NB [62]. KIRs have also been demonstrated to interact directly with MHC class I molecules. Genotyping studies from 169 patients with INSS stage 4 NB undergoing hematopoietic stem cell transplantation showed that patients with the phenotype lacking MHC class I ligands for autologous inhibitory KIR were associated with better clinical outcomes. Of note, this association was stronger than that of *MYCN* amplification [63]. Similarly, KIR/MHC class I mismatch was associated with superior treatment responses in patients with relapsed/refractory NB who were treated with an antibody against GD2 that was linked to human IL-2 [64]. Finally, treatment of INSS stage 4 NB patients with murine anti-GD2 monoclonal antibody 3F8, GM-CSF, and retinoic acid improved progression-free survival (PFS) by 18% and increased overall survival (OS) by 32% compared to the administration of the 3F8 antibody alone [65].

Functional cross-talk of natural cytotoxicity receptors (NCRs), specifically NKp46, NKp44, and NKp30 triggers NK activation via its association with immunoreceptor tyrosine-based activation motif (ITAM)-bearing signaling molecules, type I trans-membrane-anchored proteins FcεRI-γ, CD3-ζ, and DAP12, counterbalancing the inhibitory signals [66, 67]. The association of activating receptors with ITAM motif adaptors is a common feature of activating receptors, including CD16, which together with NKp46 and NKp30 associates with FcεRI-γ and CD3-ζ, while NKp44, KIR-S (activating isoforms of KIR receptors), and CD94/NKG2C are coupled with DAP-12 [68, 69]. CD16 (Fc gamma receptor IIIa) is pivotal in orchestrating ADCC through the interaction with the constant region of antibodies already bound to tumor cells [70]. Early studies investigating the role of NK cells in NB showed that blocking of NCRs (NKp46, NKp44, and NKp30) led to inhibition of NB cell line killing [71]. Moreover, the expression of NKp30 isoforms was linked to 10-year event-free survival in high-risk NB cohorts, and serum levels of B7-H6, a ligand of NKp30, correlated with loss of NKp30 expression, bone marrow metastases, and chemoresistance [72].

The C-type lectin-like activating receptor NKG2D (also known as CD314 and KLRK1), despite being a member of the NKG2 family, displays minimal genetic similarity to NKG2A and NKG2C, which are likely derived from gene duplication and lack of dimerization with CD94 [56]. NKG2D activation in humans induces potent killing activity of NK cells via the interaction with the short transmembrane molecule DAP10, which features a tyrosine-based motif distinct from ITAMs [73] (Fig. 1). MHC class I-related chain A/B (MICA/B) and UL1-6 binding proteins (ULBPs) can serve as ligands for NKG2D [74] and have been described in the context of NB (Fig. 1). While mRNA expression for all the ligands was present in most primary NB specimens and cell lines, MICA protein expression was absent in primary tumors and only present in some of the NB cell lines. Moreover, the soluble form of MICA was detected in the serum of most patients, and it was linked to decreased NK cell-mediated killing of MICA-expressing NB cells through the downregulation of surface NKG2D expression [47]. While ULBP-2 was expressed by nearly half of the NB tumors, ULBP-1 and ULBP-3 expression was generally absent from primary tumors and was expressed in about half of the NB cell lines [47]. Finally, expression of NK activating receptors, including DNAM-1, CD16, NKG2C, CD94, and NCR1, which positively correlate with genes associated with NK cytotoxicity, was lower in NB compared to expression in blood NK cells [75], and reduced expression of NKG2D and DNAM-1 was dependent on *MYCN* amplification [76].

Studies investigating the poliovirus receptor (PVR), which is expressed on NB cells and is recognized by DNAM-1 [77], showed PVR-mediated cytolysis on patient-derived BM aspirates [77], albeit to a lesser extent than when compared to the cell line model, with ex vivo PVR expression inversely correlating with *MYCN* amplification [76]. Moreover, tumor cells isolated from patients at the onset of the disease expressed PVR, whereas patients experiencing disease relapse lacked expression of PVR [77], indicating a possible link between PVR expression and disease progression. Newer studies employing monoclonal antibodies against either DNAM-1 or PVR resulted in strong inhibition of tumor cell cytolysis [78]. Therefore, it is conceivable that NB tumors that lack MHC class I expression along with lack of or diminished expression of PVR can easily circumvent the cytotoxic effects of both T and NK cells, respectively. Another ligand of DNAM-1 is Nectin-2, which is closely related to PVR [79]. The expression of Nectin-2 is inversely correlated with survival outcome in NB patients [80]. A recent study employing single cell data from primary NB patients explored cell–cell interactions between tumor and immune cells, and identified NECTIN2/TIGIT as a pivotal immune checkpoint axis in NB, which leads to dysfunctional T and NK cells [75]. Moreover, double immune checkpoint blockade of TIGIT and PD-L1 in three different NB murine models (N1E-115, Neuro2a, and N18) led to superior survival outcomes compared to single treatment with PD-L1 [75].

NK cell activation mechanisms extend beyond cytotoxic effector functions, they also include the release of cytokines and chemokines to alert and recruit other immune cells. Earlier studies investigating the coordination of these responses showed that the triggering of single receptors, including CD16 and NKG2D, induced rapid secretion of chemokines, while the secretion of cytokines, such as TNFα and IFNγ, required activation of several different receptors [81]. NK cells are one of the primary producers of IFNγ, which has been shown to significantly enhance the cytotoxic activity and tumor infiltration of NK cells. It also has the ability to boost the immune-promoting functions of T cells, conventional dendritic cells, and B cells. However, IFNγ exposure can also induce immunosuppressive phenotypes, such as regulatory T cells (Tregs) and exhausted T cells, ultimately leading to immune evasion and tumor growth [82, 83]. A study investigating IFNγ effects on NK-mediated lysis in 22 pediatric tumor cell lines, including NB, revealed varied responses to treatment, with some NB lines remaining unaffected, some developing resistance, and others becoming more sensitive [83]. These findings highlight the dual pro- and anti-tumorigenic roles of IFNγ, and the significant variability among NB cell lines, likely reflecting the clinical heterogeneity of NB.

As described above, the mechanisms by which NK cells recognize various motifs to execute regulatory functions are quite complex and are just beginning to be understood. With advancement in technologies and treatment modalities, the abovementioned proteins may emerge as targets for small molecule immunomodulators that may have therapeutic benefits in NB.

### NK cell infiltration and prognostic value in NB

Across various tumor types, higher presence of infiltrating NK cells has been positively associated with prognosis [84], and this paradigm holds true in NB. Several studies have reported that presence of NK cells varies between low-risk and high-risk disease, with increased levels of NK cells correlating with favorable outcomes [20, 85–87]. One study employing flow cytometry characterized the immune cell type composition in nine patients with INSS stage 4 NB and demonstrated that NK cells comprise 1–30% of the total cells isolated [88].

The data underscoring the association between *MYCN* amplification and NK cell infiltration are contradictory. Initial studies have reported that tumor infiltration by NK cells was dependent on *MYCN* amplification, with *MYCN* non-amplified patient samples marked by higher infiltration [85, 89]. However, this higher infiltration was not linked to increased cytotoxicity, as noted in a large cohort study of 498 primary NB patient-derived samples [90]. Moreover, expression of key genes related to the effector functions of NK cells is reduced in *MYCN* amplified NB patients compared to *MYCN* non-amplified cases [85]. A recent study employing single-nucleus RNA-sequencing showed lack of NK cell infiltration in pre-treated primary NB, but presence of NK cells in the same patient following treatment and resection of the tumor [91]. Additional studies in primary and metastatic NB with or without *MYCN* amplification using single-cell RNA-sequencing showed that while NK cell infiltration can also be high in intermediate- and high-risk NB with amplified *MYCN* [92, 93], the presence of CD56^dim^ NK cells was not linked to improved clinical outcome [92]. On the other hand, enrichment for active NK cells in primary NB as defined by expression of immediate-early genes, including *ZPF36*, *NR4A2*, *JUNB*, *FOS*, *FOSB*, and *CD69* was associated with improved outcome, but this significance did not hold true when the data were separated by risk group and *MYCN* status [92].

Therefore, it is unclear to which extent *MYCN* amplification affects the disbalance in NK cell populations and how NK cell infiltration alone contributes to disease outcome.

## Tumor escape mechanisms

**Fig. 2:**
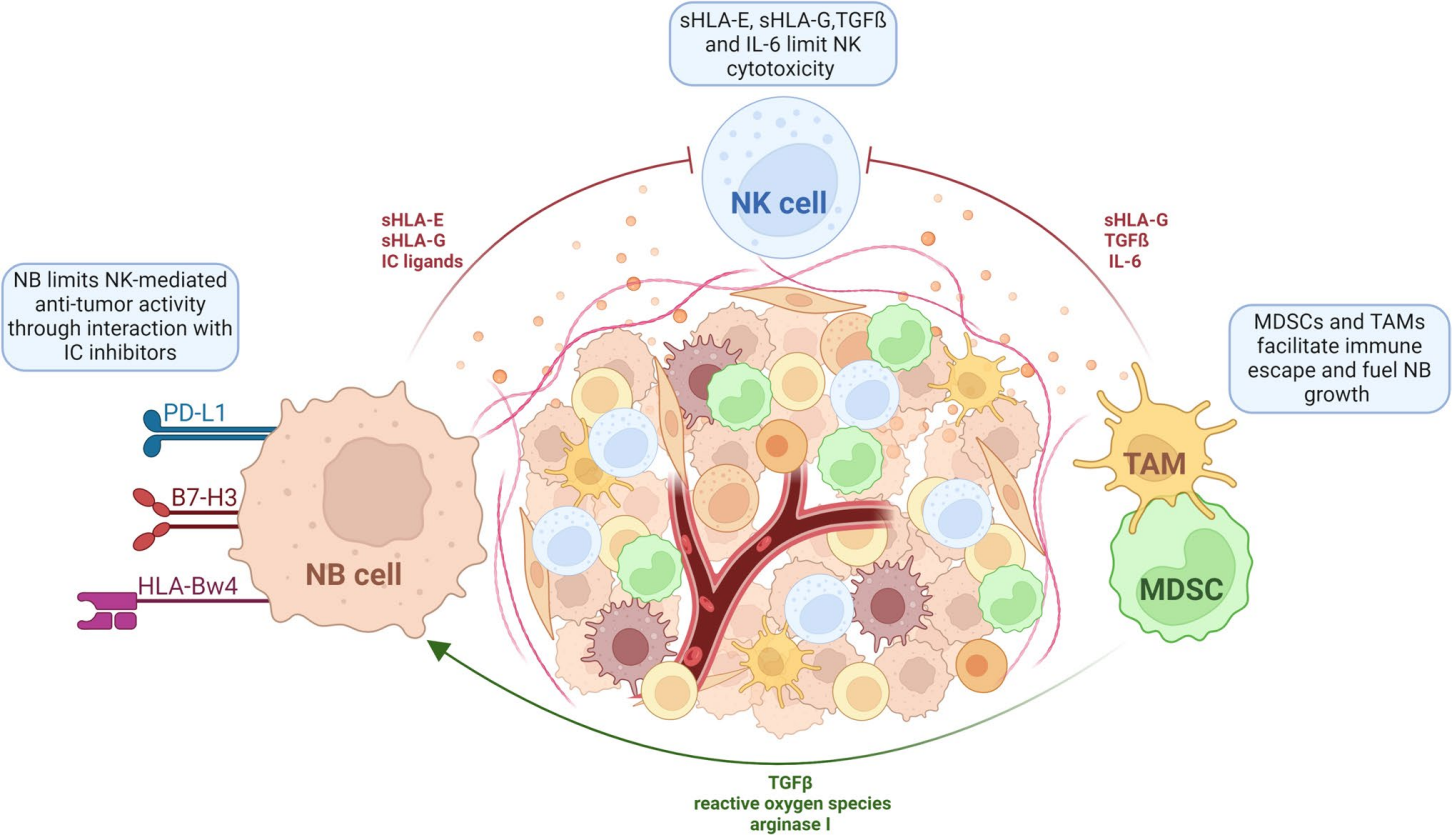
Suppressive dynamics within the NB TME. Myeloid-derived suppressor cells (MDSCs) and tumor-associated macrophages (TAMs) in the NB tumor microenvironment (TME) release immunosuppressive agents, including TGFβ and IL-6, which reduce the cytotoxic capabilities of NK cells. Simultaneously, TGFβ, reactive oxygen species, and arginase I, promote NB progression. Additionally, NB cells emit further signals that hinder NK cell cytotoxicity through the release of soluble (s)HLA-E and sHLA-G molecules. sHLA-G can also be produced by monocytes upon interaction with NB cells, further attenuating NK cell killing. NB cells also express ligands like PD-L1, B7-H3, and HLA-Bw4, which engage inhibitory immune checkpoint (IC) receptors on NK cells, facilitating NB evasion from NK cell-mediated eradication. Figure was created with BioRender.com

Tumors have evolved to evade immune surveillance through an array of activities that suppress NK cell-mediated killing as discussed in Sect. 2.1. Additionally, the poor ability of NK cells to reach solid tumors along with the immunosuppressive milieu produced by the TME all lead to dampened activity of NK cells through the secretion of various cytokines and soluble factors [44] (Fig. 2). The current battle in immunotherapeutics is to understand the underlying mechanisms that lead to these adaptations and how this impacts NK cell responses.

### Bidirectional interactions between NK cells and the tumor microenvironment

The TME is defined as the interplay between cellular and molecular components, namely tumor, stromal, and immune cells, along with soluble factors, which altogether contribute to an immunosuppressive niche. This, in turn, allows the tumor cells to evade immunosurveillance and subsequent elimination by NK cells. Several studies have established the roles of immunosuppressive molecules, such as TGFß and IL-6 in modulating the TME and dampening NK cell-mediated cytotoxicity [47, 75, 94, 95]. For instance, TGFß has been shown to downregulate the expression of activating receptors NKG2D and NKp30, and adaptor proteins DAP10 and DAP12 [96–98]. Additionally, TGFß can mediate transdifferentiation of NK cells into type 1 innate lymphoid cells [99], which lack cytotoxic capabilities. Studies employing genetically modified NK cells that were armed with variant TGFß receptors showed increased cytotoxicity against NB cells in vitro and improved progression-free survival in vivo [100]. Blockage of TGFß in co-cultures of NB cells with NK cells derived from healthy donors resulted in increased cytotoxicity, whereas addition of recombinant TGFß1 led to reduced tumor killing and decreased production of granzyme B and was marked by dysfunctional NK cells [75]. Reduced expression of TGFB1, a ligand for the TGFß signaling pathway, was linked to retinoic acid therapy resistance in *MYCN* amplified cell lines [101], whereas TGFB1 upregulation in primary NB patients without *MYCN* amplification was associated with worse clinical outcome [102].

Co-culturing suppressive macrophage populations with NK cells dampened the effector functions of NK cells via TGFß and IL-6 production, reduced the release of cytotoxic proteins, granzymes A and B, downregulated expression of perforin, and suppressed IFNγ secretion [103]. Treatment of nonobese diabetic/severe combined immunodeficiency (NOD/SCID) mice bearing NB tumors with an injection containing NK cells, anti-GD2 antibody, and lenalidomide was superior than the treatment without lenalidomide, with the latter blocking IL-6 and TGFβ1 signaling, thus overcoming the suppression of NK cells [103]. High levels of IL-6, noted in tissue and blood samples of NB patients, have been associated with poor 5-year event-free survival [102, 104]. Of note, IL-6 has been demonstrated to be stimulated by galectin-3 binding protein, which is secreted by NB cells, thereby raising the thought that this is an autoprotective mechanism gained by tumor cells to evade surveillance [105–107].

Tumor cells also release additional immunosuppressive molecules, including soluble (s)HLA-E and sHLA-G. Upregulated expression of HLA-E in NB was associated with disease stage as well as *MYCN* status. In vitro experimental studies have shown that upregulated HLA-E expression leads to reduced NK cell cytotoxicity, an effect mediated by induced IL-10 and TGFß production [108]. Another study reported increased levels of sHLA-G produced by monocytes in response to NB cells, and this resulted in attenuated function of NK cells [109]. Moreover, levels of both sHLA-E and sHLA-G were higher in metastatic NB than in primary NB, where serum levels of HLA-G in NB patients were prognostic markers for disease relapse [109, 110], suggesting a role in disease progression (Fig. 2).

Other cell communities within the TME, such as myeloid-derived suppressor cells (MDSCs) and tumor-associated macrophages (TAM), attenuate the effector function of NK cells, with MDSCs accumulation being linked with tumor progression in a TH-*MYCN* transgenic mouse model [111]. Furthermore, MDSCs fuel tumor growth by producing TGFβ, reactive oxygen species, and arginase I [112] (Fig. 2). L-arginine is catabolized by arginases I and II to produce metabolites, such as ornithine and urea, which play central roles in facilitating tissue healing and remodeling. Conversely, its catabolism by inducible nitric oxide synthase generates nitric oxide (NO), which induces cytotoxicity and inflammation [113]. In the TME, macrophages polarize to an immunosuppressive M2 phenotype, characterized by arginase I-mediated cytoplasmic hydrolysis of L-arginine, resulting in reduced NO production due to decreased intracellular L-arginine availability [114]. While this mechanism serves to prevent excessive inflammatory tissue damage in healthy conditions, it is exploited in NB to facilitate tumor formation and progression [115]. The depletion of L-arginine inhibits the proliferation and cytokine secretion in T and NK cells, fostering an immunosuppressive environment [116, 117]. While MDSCs and M2 TAMs predominantly express arginase I, NB cells, both from patients and TH-*MYCN* transgenic mice express predominately arginase II, and this was associated with poor clinical outcome in NB patients [118, 119].

The tumor microenvironment is extremely complex, with various components ultimately serving to suppress the immune surveillance to promote tumor growth. Further characterization of the complex interplay between NK cells and the various cellular communities and molecular factors encompassing the TME may elucidate new targetable interactions and provide a basis for new therapeutic approaches.

### The role of immune checkpoint molecules in suppressing NK cell activity

Immune checkpoint (IC) proteins expressed on the surface of most immune cells allow them to regulate their responses post activation and monitor for illicit proliferation. These proteins can be broadly categorized as inhibitory and activating receptors, where the first serve as immune checkpoints, transmitting inhibitory signals that help maintain peripheral tolerance and prevent autoimmunity [120, 121]. Most tumor cells, including NB, have high expression of ligands that bind to the inhibitory receptors, allowing for tumor cells to go undetected during the initial stages of the disease. These also act as a major barrier in cancer immunotherapy, often leading to tumor unresponsiveness during treatment. Several newly identified immune checkpoints, including PD-1, KIRs, and TIGIT are pertinent to nearly all immune cells [122–128].

The programmed death-ligand 1 (PD-L1) binds to PD-1, which under normal settings informs NK cells, amongst others, to shutdown their immune response [129], preventing harm to healthy cells. In NB cell lines, expression of PD-L1 appears to be independent of the *MYCN* amplification status and is constitutively expressed in cell lines positive for MHC I [130]. Another study in NB patients reported that PD-L1/MHC I expression can be employed as marker to predict overall survival however, PD-L1 expression was regulated by *MYC* and *MYCN* [131]. The expression of PD-1 receptor in metastatic NB was noted to be confined to αβ T cells and to a lesser extent in γδ T cells and NK cells [130]. Immunohistochemical analysis of 31 NB patients demonstrated PD-L1 expression in 11 samples, and this population was associated with decreased survival [132]. Additional studies investigating the role of the PD-1/PD-L1 axis in NB demonstrated a large variation in PD-L1 expression, where decreased survival and increased risk of relapse was noted in high-risk and INSS stage 4 NB tumors that expressed PD-L1, whereas no differences in clinical outcome were noted in more differentiated tumors graded as INSS stage 1/2/3 or those with low- and intermediate-risk [133, 134]. While it is evident that PD-1 and PD-L1 are expressed in NB (Fig. 2), it is unclear how heterogenous the expression within and across patients might be, as such their contribution to disease progression remains to be investigated.

Another IC inhibitor that has been reported to be relevant in suppressing NK cell effector functions is B7-H3, however, the specific receptor(s) that it binds to on NK cells have yet to be identified. In NB patients of all stages, high expression of B7-H3 was linked to overall worse event-free survival [135], whereas decreased expression of B7-H3 was linked to better clinical outcomes [136]. In addition, overexpression of B7-H3 protects NB cells from NK-mediated cytotoxicity and in vitro inhibition of B7-H3 resulted in enhanced NK cell-mediated cytotoxicity [137] (Fig. 2).

KIRs are IC inhibitors that interact with MHC class I molecules and use of a humanized GD2 antibody (3F8) led to activation of NK cells lacking HLA-Bw4 ligand for KIR3DL1 [138]. This was dependent on the strength of interaction between KIR3DL1 and HLA-B subtypes and was associated with therapeutic response to anti-GD2 and better patient outcome [139]. Moreover, co-cultures of NB cells expressing HLA-Bw4 and NK cells lacking KIR3DL1, but expressing KIR3DS, a receptor for KI3DS1 on NB cells led to NK cell-mediated cytotoxicity, and this was linked to improved progression-free and overall survival (Fig. 2) [139].

The abovementioned IC inhibitors appear to negatively affect the role of NK cells in NB, thereby attenuating their anti-tumor activity. Future studies will have to disaggregate the roles of these IC inhibitors in different NB subtypes and demonstrate how they will benefit patients.

## Therapeutic opportunities

In this section, we consider how our understanding of the mechanisms that lead to NK cell activation/inactivation as well as the strategies that tumor cells employ to evade NK cell cytotoxicity can be harnessed as immunotherapies. Such therapies involve, first, the activation of endogenous NK cells through antibodies directed against tumor-specific antigens, e.g., GD2, stimulation with cytokines and, second, in vitro engineering and expansion of NK cells as cellular therapeutics.

### Enhancing endogenous NK cell effector functions

NK cells require the presence of cytokines, namely interleukins, to enhance their cytotoxic effect by promoting their proliferation, survival, and activation. The role of interleukins (IL-2, IL-12, IL-15, IL-18, and IL-21) as immunostimulatory molecules is well documented in the literature and has been noted to promote proliferation of NK cells, both in vitro and in vivo [140, 141]. Infusion of pretreated NK cells with IL-2 into NOD/SCID mice bearing metastatic human NB cells resulted in reduced BM infiltration and increased mean survival, with an augmentation of this effect by the administration of IL-2 and IL-15 [142]. Earlier studies in INSS stage 4 NB patients that were in complete/partial remission who were administrated high doses of IL-2 showed a biphasic response: in the initial stages of the treatments, there was an increase in NK cell proliferation, followed by increased NK cell-induced cytotoxicity during the later stages [24]. However, the median follow-up of patients was only 24 months, thus, duration of treatment response was not evaluated. Several other studies demonstrated no benefits of IL-2 therapy in NB patients [143, 144]. Of note, the adverse effects of high IL-2 doses are not fully understood, however, systemic effects, including vascular leak syndrome, heart failure, and liver failure, have been observed [145]. Immune disturbances secondary to stimulation of Tregs have also been reported, which would counteract the benefits arising from the stimulation of NK cells [146]. Given the high dose IL-2-associated toxicities (administered in combination with other treatment modalities) and unknown roles of low dose IL-2 in NB, use of IL-2 for treatment of NB has been discontinued [143].

Treatment with recombinant anti-GD2 (ch14.18)-IL-2 fusion proteins have showed promising results and led to NK cell-mediated eradication of NB tumors in the BM and liver metastases in a murine syngeneic model [147]. Similarly, administration of recombinant anti-GD2-IL2 along with GM-CSF and isotretinoin was beneficial for high-risk and relapsed/refractory NB patients [33, 35, 148]. This effect is believed to be through the activation of ADCC and complement-dependent cytotoxicity, respectively, leading to both, NK and T cell action through IL-2 receptor binding [149–151].

Combinatorial treatment with high doses of chemotherapy in tandem with autologous hematopoietic stem cell transplantation (ASCT) has shown promising results for high-risk NB patients and is now part of a standard treatment regimen in the USA [152–155]. On the other hand, in case of haplo-stem cell transplantation or allogeneic transplant, T cell depletion is required to prevent GvHD, and in their absence, NK cells play a key role in eliminating residual tumor cells [12]. In vitro studies have reported that PBMC-derived NK cells following stimulation with IL-2, IL-15, and/or IL-21, and depletion for alloreactive CD3/CD19 T cells, expanded faster than cytokine-induced killer (CIK) cells, and NK cells showed higher in vitro cytotoxicity against NB cells than CIK [156]. Similarly, ex vivo expanded PBMC-derived NK cells, upon simulation with IL-15, demonstrated increased viability and proliferation, as well as increased cytotoxicity against various solid tumors in mice, including NB [157]. Preclinical studies performed in vitro and in mouse models with orthotopic NB have demonstrated that integration of IL-15 [158] and IL-21 [159] in a NB immunotherapy regimen (with anti-GD2 and GM-CSF) exhibits anti-tumor activity and outperforms the anti-GD2-IL-2 combination [159]. Finally, NK cells derived from healthy donors and NB patients that were ex vivo stimulated with IL-2 and/or 562-mbIL21 stimulatory cells were injected into NB murine models, along with anti-GD2 [160, 161] and/or IL-2, and IL-15 [160, 162] before and after tumor resection [160]. Mice that received the therapy before surgery had better clinical outcomes than the ones that received it post tumor resection [160]. While these treatments have shown promising results, the severe side effects attributable to the use of interleukins as well as the high rates of disease relapse, highlights the need for improved treatment approaches [163].

The combination of TGFβ1R1 inhibitor, Galunisertib (LY2157299) with Dinutuximab and NK allogeneic infusion seems to be a promising therapeutic option. A study demonstrated enhanced NK cell-mediated cytotoxicity in mice xenografted with NB cell lines or patient-derived NB, and was marked by restored expression of TRAIL, DNAM-1, NKG2D, and NKp30 [164]. Thus, targeting TGFβ1R1, the most commonly upregulated member of the TGFβ family with Galunisertib might offer a promising strategy to enhance the anti-NB efficacy of Dinutuximab combined with adoptively transferred activated NK cells [164]. This approach might be superior to targeting TGFß signaling itself, given the dual nature it plays in cancer and its crosstalk with TME, which has resulted in challenges in developing therapeutic agents for use in cancer [165, 166]. Emerging approaches suggest combining TGFβ inhibitors, such as TGF-βRII, as a decoy receptor with PD-L1 antibodies, allowing for inhibition of TGFβ within the TME [167]. Other novel therapeutic avenues include a glycosylphosphatidylinositol anchor, dominant negative for TGF-ß receptor II, which could be combined with other immune checkpoints [168]. These approaches are just beginning to be investigated in NK cells, thus, their outcome in preclinical and clinical settings remains to be understood.

### Adoptive NK cellular therapy

Adoptive transfer of cellular products is an up-and-coming therapeutic strategy that holds tremendous promise. Initial studies in human trials reported that adoptive transfer of haploidentical NK cells does not impact the engraftment of neutrophils and platelets, and that the toxicities were comparable between patients with and without NK cell infusion [169–171]. Similarly, other studies showed that haploidentical infusion of NK cells without ex vivo expansion led to increased NK cell-mediated cytotoxicity during treatment as compared with time at diagnosis [22], and in one case, a complete remission was observed [172]. Adoptive NK cell therapy along with m3F8, an anti-GD2 antibody, but with higher affinity than Dinutuximab, led to a complete or partial response in 29% of the patients, 47% did not respond to the treatment, and 23% were characterized by progressive disease. A proposed mechanism for this effect was through increased NKG2A expression [173]. As noted above, the mismatch in KIR ligands has proven valuable in these treatment approaches [63]. A trial was conducted, where patients were infused with parent-derived NK cells along with treatment with humanized ch14.18K322A (and chemotherapy, IL-2, and GM-CSF), and another anti-GD2 antibody with similar affinity to GD2 as Dinutuximab, resulted in a complete or partial response in 61.5% (8/13) of patients, with the remaining noted to have stable disease [23]. Various clinical trials at different stages are currently ongoing or have been completed (Table 1) with the aim to determine the best dosage, efficacy, and combinatorial treatment when including NK cell infusions.

**Table 1:** Completed and ongoing clinical trials for adoptive NK cell therapy in combination with other treatment regimens in neuroblastoma

T**reatment**	**Clinical trial phase**	**Status **	**Clinical trial ID**
Anti-GD2 (ch14.18/CHO) + expanded and activated haploidentical NK cells at escalating dose levels	Phase I/II	Unknown status	NCT03242603
Cyclophosphamide, Fludarabine, and Mesna + haploidentical donor-derived, IL-2-activated NK cells + low-dose IL-2	Phase II	Terminated	NCT00698009
Autologous NK cells activated and expanded ex vivo using artificial antigen-presenting cells (aAPCs) expressing human 4–1BBL and human IL-15Rα	Phase I	Completed	NCT01875601
Autologous NK cells at escalating dose levels + Dinutuximab, with and without Lenalidomide	Phase I	Active, not recruiting	NCT02573896
HLA-haploidentical hematopoietic cell transplantation + early post-transplant donor NK cell infusion	Phase II	Completed	NCT02100891
Ex-vivo expanded allogeneic universal donor TGFβi NK cells + Irinotecan, Temozolomide, Dinutuximab, Sargramostim	Phase I/II	Recruiting	NCT04211675
Donor-derived, IL-15 and 4-1BBL-activated NK cells + HLA-matched, T-cell-depleted nonmyeloablative peripheral blood stem cell transplantation	Phase I	Completed	NCT01287104
Cyclophosphamide + hu3F8 + NK cell infusion + subcutaneous rIL-2	Phase I	Active, not recruiting	NCT02650648
HLA-haploidentical HSCT + CD3-depleted/CD^56+^ selected NK cells from apheresis products	Phase I/II	Completed	NCT01386619
Tumor-specific standard chemotherapy (Cyclophosphamide, Topotecan, Temozolomide, Irinotecan, Carboplatin, Ifosfamide, Etoposide) + humanized anti-GD2 antibody (hu14.18K322A), with/without haploidentical NK infusion	Phase I	Completed	NCT01576692
Intravenous combinational chemotherapy (Cyclophosphamide, Vincristine, Topotecan) + allogeneic NK cells from HLA-haploidentical related donor + 3F8 infusions	Phase I	Completed	NCT00877110
High-dose chemotherapy (Busulfan, Melphalan + CD^133+^ selected allogeneic stem cell infusion + hu14.18K322A + IL-2 + haploidentical NK cells + G-CSF + GM-CSF	Phase I	Completed	NCT02130869
Fludarabine, Busulfan IV, TBI 2 grays, and CD3/CD19 graft depletion followed by HSCT + CD^56+^ NK cell injections	Phase II	Unknown status	NCT01156350
HSCT + ex vivo expanded NK cell infusion and low-dose IL-2	Phase II	Unknown status	NCT01807468
hu14.18K322A + chemotherapy (Cyclophosphamide, Topotecan, Cyclophosphamide, Coxorubicin, Vincristine, Cisplatin, and Etoposide) with Mesna given before and after Cyclophosphamide infusion + peripheral blood stem cell harvest and surgical resection of the primary tumor + Busulfan, Melphalan, and Levetiracetam with peripheral blood stem cell transplantation and radiation therapy + hu14.18K322A with allogeneic NK cell infusion + hu14.18K322A, G-CSF, GM-CSF, interleukin-2, and isotretinoin	Phase II	Active, not recruiting	NCT01857934
HLA-haploidentical familial donor bone marrow transplantation + intravenous NK cells at escalating levels	Phase I	Completed	NCT00569283

Another approach to adoptive transfer involve the CAR-engineered NK cells that via genetic modifications are armed with synthetic receptors, which increase the specificity as well as the efficacy of NK cells against their targets. There have been many generations of CARs, but in essence, they are fusion proteins and their structure generally consists of three components: the extracellular antigen-binding domain (usually an scFv), the spacer, and the transmembrane intracellular domain [174, 175]. Although still under clinical evaluation, CAR-NK cells offer significant advantages over CAR-T-based therapies. These advantages include a shorter lifespan, mitigating the risk of overexpansion, the secretion of safer cytokines, such as IFN-γ and GM-CSF, and the capacity to lyse target cells through both CAR-dependent and independent mechanisms [141]. Additionally, the low risk of NK cell rejection [176, 177] allows for CAR-NK cells to be derived from various sources, including PBMCs, umbilical cord blood, bone marrow, stem cells, and even NK cell lines, such as NK-92 [178–180]. It is relevant to note that NK-92 cell line was originally derived from a leukemia patient, and thus, the product needs to be irradiated before being administered into patients to prevent any potential tumor formation [181]. However, despite these promising benefits, CAR-NK therapy has encountered several critical challenges. Recent studies have highlighted distinct costimulatory signal requirements for NK-92 cell lines, primary NK cells, and hPSC-derived NK cells, highlighting the necessity for tailored CAR structures to optimize NK-CAR function and minimize off-target toxicity [182, 183]. Moreover, enhancing transduction methods is crucial to improve efficiency and mitigate viral vector genotoxicity [174, 184]. Improvement of the purification protocols for allogeneic NK cells will also be essential to prevent contamination by T or B cells, thereby reducing the risks of GvHD and lymphoproliferative disorders [185–187].

Several CAR-NKs have been developed and tested in NB. For instance, NK-92 cells transduced with a CAR armed with anti-GD2 scFv as well as CD3ζ domains showed high cytotoxicity against NB cells that were positive for GD2, whereas these cells were resistant to the parental NK-92 cell line [26]. Another study generated CAR-NK cells derived from NB patients bearing activating receptor NKG2D fused with the cytotoxic ζ-chain of the T-cell receptor and demonstrated that these CAR-NK cells are cytotoxic against MDSCs, while tissues expressing NKG2D remain unaffected. Elimination of MDSCs, in turn, allowed for more efficient infiltration of chemokines and cytokines, and tumor cytotoxicity by CAR T cells [188].

However, challenges remain, in the context of GD2 directed CAR-T and NK cell therapy. For instance, expression of indoleamine-pyrrole 2,3-dioxygenase1 (IDO1) significantly impairs, both T and NK cell activity by depleting tryptophan and generating immunosuppressive kynurenines. This, in turn, impairs IFNγ secretion in NB cells, thereby rendering these cells resistant to NK cell-mediated cytotoxicity [189]. A recent study identified aryl hydrocarbon receptor as a receptor for kynurenine, which might prove as a useful target to circumvent the IDO1-associated attenuation of NK cell effector functions [190]. GD2-mediated ADCC is inhibited by the presence of mesenchymal stromal cells (MSC), monocytes, and endothelial cells, all of which are CD105 + . The use of TRC105 (an anti-CD105 antibody) in primary NB cell lines or combined with Dinutuximab and activated NK cells in NB mice models, restored ADCC-mediated cytotoxicity, and effectively eradicated the tumors [191, 192]. These effects were counteracted in the cell lines following the addition of kyrenine, among others, again highlighting the relevance of IDO1 in NK cell function and ADCC [192]. Use of a dual inhibitor IDO1/TDO (RY103) effectively suppressed IDO1 in a pre-clinical murine pancreatic cancer model [193] and might be a new avenue to be considered in circumventing the inhibitory action of IDO1 in NK cell-mediated cytotoxicity.

While progress has been made in experimental models, future endeavors should be focused on identifying ways to improve the effector functions and persistence of CAR-NKs. Another approach to enhance NK cell-mediated cytotoxicity involves the use of bi-specific and tri-specific killer cell engagers (BiKEs and TriKEs). These engineered molecules consist of the variable heavy and light chains, targeting both tumor-associated antigens and NK cell-activating receptors, connected by a short flexible polypeptide linker, facilitating the formation of an immunologic synapse between NK and tumor cells [194]. This treatment avenue has proven promising in various cancer cell lines. For instance, 16133 BiKE, consisting of scFv binding FcγRIII (CD16) on NK cells and CD133 as a base, created 1615133 TriKE by addition of IL-15. This led to increased NK cell-mediated cytotoxicity and greater NK cell expansion compared to the BiKE lacking IL-15 in a host of cancer cell lines: colorectal cancer, Burkitt lymphoma, and promyelocytic leukemia cell lines [195, 196]. Moreover, a TriKE to target acute myeloid leukemia (AML) was developed to contain a humanized anti-CD16 heavy chain camelid single-domain antibody, which activates NK cells, an IL-15 molecule that drives NK-cell expansion and survival, and an scFv against human CLEC12A that is highly expressed in AML [197]. This led to successful killing of AML cell lines and primary patient-derived AML blasts, but did not affect the hematopoietic stem cells, highlighting its specificity [197]. Another TriKE, which contained a camelid anti-CD16 antibody fragment, a wild type IL-15, and an anti-B7-H3 scFv was tested in vitro in an array of cancer cell lines as well as in NSG mice grafted with MA-148 ovarian cancer cell line. This, in turn, resulted in NK cell expansion, specific killing of tumors expressing B7-H3, and led to reduced tumor growth in vivo [198]. Given the benefits of IL-15 [156–158] as well as the overexpression of B7-H3 [135–137] in NB patients, it might be relevant to pursue these immunotherapeutics in preclinical trials for this tumor.

### Immune checkpoint inhibitors

Immune checkpoint blockade uses monoclonal antibodies to target inhibitory checkpoints on immune cells or their ligands expressed by tumor cells [141]. They aim to unblock suppressed immune responses and attempt to reverse functional inhibition by disrupting these receptor/ligand interactions, restoring effective anti-tumor cytotoxic activity, and potentially leading to lasting tumor regression. Blocking inhibitory receptors can restore the cytotoxic activity of NK cells against cancer cells [141].

There are currently several anti-PD1 and anti-PD-L1 antibodies that have gained approval for treatment of solid malignancies, but none have yet gained approval for use in NB. However, several experimental trials have demonstrated their successful application against NB. The combinatorial treatment of anti-GD2 and anti-PD1 resulted in increased overall survival in a lethal syngeneic murine model engineered using *MYCN*- and TH-positive NXS2-HGW NB cells [199]. A similar response was observed in two patients with relapsed/refractory NB, where the combined anti-GD2/anti-PD1 treatment resulted in a complete remission and a partial remission, respectively [28]. Dual immune check point blockage by anti-CTLA-4 and anti-PD1 in syngeneic mice with NB tumors, resulted in tumor regression and improved OS, which was attributed to increased levels of T cells, NK cells, and inflammatory macrophages [200]. In vivo studies combining an antagonist for colony-stimulating factor 1 receptor (CSF-1R) with anti-PD1 resulted in better control of tumor growth [201], while in vitro studies showed that this was mainly mediated by hampering activation of suppressive myeloid cells and by reinvigoration of T and NK cells [202].

Treatment with single agent, CTLA-4 antibodies in mice bearing GD2 expressing NB tumors (NXS2 or 9464D-GD2) did not result in reduced tumor volume, however, the combinatorial treatment with anti-CD40 monoclonal antibody and CpG-oligodeoxynucleotides greatly improved immune response by reinvigorating T cells and their response, and by reducing Treg population, but no differences in NK cells were found [203]. Treatment of syngeneic mice bearing synchronous Neuro2a tumors with CpG-coated Prussian blue nanoparticles-based photothermal therapy and anti CTLA-4 immunotherapy was characterized by complete tumor regression in both primary and secondary tumors, as well as improved rates of long-term survival compared to controls, and this was dependent on activation of CD4 + and CD8 + T and NK cells [204]. Finally, combinatorial treatment with anti-CTLA-4 and survivin-derived peptide cancer vaccine, where the latter is characterized by high affinity for the murine analog of MHC-encoded class I molecule (H2-KK) [205], led to abrogation of tumors in mice bearing AgN2a and NXS2 NB tumors, and this was mediated by CD8 + T and NK cells [206]. The role of these inhibitors, which seem promising in murine models, is yet to be established in the context of patients with NB, but in the future, may prove to be promising avenues.

## Conclusions and perspectives

To date, extensive efforts have been directed towards better understanding the role of NK cells in NB and how to harness the cytotoxic capabilities of NK cells into therapeutic modalities. While there are several ongoing experimental studies, apart from anti-GD2 antibody therapy, few NK-mediated therapies have reached clinical platforms, with the immediate challenge being low efficacy in clinical trials. NK cell tissue-specificity [207–209] along with the heterogenous nature of NB adds a further layer of complexity into NK cell-mediated therapies in NB, and posits the question whether future cellular therapies would need to be designed specifically against primary and metastatic NB, given the stark mechanistic differences observed amongst different high-risk NB subgroups. This, in turn, would also serve as a path into investigating the relationship between immune diversity and immunotherapeutic response. Our understanding of the TME is evolving quickly, and these findings provide a powerful platform to continue to identify strategies that will hopefully enable us to efficiently override the immunosuppressive TME and avoid dampening of NK cell activity. However, the functional roles and therapeutic potential of many established NK ligand-receptor interactions remain largely unexplored in NB. While low mutational burden is a defining feature of NB and associated low immunogenicity, emerging studies point to other factors that might impact this constellation. For instance, NB tumors characterized by the presence of adrenergic lineages are marked by lower immunogenicity compared to the ones derived from mesenchymal lineages, and these immune gene signatures are regulated by epigenetic mechanisms [210]. While studies investigating the role of the epigenome in NK cell development and adaptive immunity [211–214] have started to gain traction, the mechanisms by which the epigenome influences the expression of key receptors/ligands in NK cell function, and how this translates into tumor evasion has yet to be understood. Focusing efforts on these fronts will, hopefully, result in emergence of NK cell therapeutic modalities to be delivered either as standalone treatment or as complementary to other treatment strategies.

## Data Availability

No datasets were generated or analysed during the current study.
